# Do Bats Have the Necessary Prerequisites for Symbolic Communication?

**DOI:** 10.3389/fpsyg.2020.571678

**Published:** 2020-11-12

**Authors:** Mirjam Knörnschild, Ahana A. Fernandez

**Affiliations:** ^1^Museum für Naturkunde, Leibniz-Institute for Evolution and Biodiversity Science, Berlin, Germany; ^2^Animal Behavior Lab, Freie Universität, Berlin, Germany; ^3^Smithsonian Tropical Research Institute, Ancón, Panama

**Keywords:** symbols, indexical communication, social learning, cognitive skills, touchscreen, training paradigm, bats, associative learning

## Abstract

Training animals such as apes, gray parrots, or dolphins that communicate *via* arbitrary symbols with humans has revealed astonishing mental capacities that may have otherwise gone unnoticed. Albeit bats have not yet been trained to communicate *via* symbols with humans, we are convinced that some species, especially captive Pteropodid bats (“flying foxes”), show the potential to master this cognitive task. Here, we briefly review what is known about bats’ cognitive skills that constitute relevant prerequisites for symbolic communication with humans. We focus on social learning in general, trainability by humans, associative learning from humans, imitation, vocal production learning and usage learning, and social knowledge. Moreover, we highlight potential training paradigms that could be used to elicit simple “symbolic” bat-human communication, i.e., training bats to select arbitrary symbols on a touchscreen to elicit a desired behavior of the human caregiver. Touchscreen-proficient bats could participate in cognition research, e.g., to study their numerical competence or categorical perception, to further elucidate how nonhuman animals learn and perceive the world.

## Introduction

Language is crucial to transmit information, share and accumulate knowledge across generations, and promote humans’ cumulative culture (Tomasello, 2000; Herrmann et al., 2007; Fitch et al., 2010). Therefore, language drives and is driven by social cognition (Tomasello, 1992; Fitch et al., 2010). Besides a large set of physical cognitive skills, language particularly requires sociocognitive skills. Physical cognitive skills include memory, categorical perception and discrimination, perceptual processing, and recognition; and some researchers would also include general learning abilities such as fast mapping or associative learning as additional prerequisites (Gopnik et al., 1999; Vihman, 2014). Sociocognitive skills include, for example, social learning and theory of mind (Tomasello, 2003; Cheney and Seyfarth, 2007; Herrmann et al., 2007; Fitch et al., 2010). A remarkable form of social learning is our ability for imitation which plays a fundamental role in speech (or sign) acquisition (Oller, 1980; Petitto and Marentette, 1991; Vihman, 2014; Fitch, 2018). Infants acquire speech through imitation of the fundamental speech subunits, i.e., syllables, based on auditory input (Oller, 1980; Vihman et al., 1986). Whereas the ability of vocal production learning, i.e., the modification of one’s own oral output based on social input, represents the mechanistic part of speech production, social knowledge is required to develop the semantic capacities of language (Tomasello, 1992, 2000; Fitch et al., 2010). The cognitive skills of joint attention, gaze responsiveness, and pointing pave the way for the developing the theory of mind in young infants (Carpenter and Tomasello, 1995; Gopnik et al., 1999; Tomasello, 2003). Joint attention, for example, is important for understanding others and enhances word learning (MacNamara, 1972; Gopnik et al., 1999; Tomasello, 2003). The development of these sociocognitive skills and, ultimately, language acquisition are shaped and promoted through social interaction (Tomasello, 1992; Kuhl, 2007; Goldstein and Schwade, 2010). Social feedback is also important for non-human vocal production learners (Goldstein and Schwade, 2010; Beecher, 2017; García, 2019), in particular, when learning non-species-specific vocalizations as the interaction in itself is already a form of communication (Pepperberg, 1992, 1994, 2002), or when learning to communicate *via* arbitrary symbols (Reiss and McCowan, 1993).

Language can be understood as a system of symbols whose elements (for example, words) can be arranged according to rules (through grammar) to create new meaningful units (such as sentences). Thus, the power of human symbolic communication is based upon the fact that the meaning of words can gain additional meaning through their relationship to other words, i.e., a sign-sign relationship (Sinha, 2004; Nieder, 2009). In contrast, non-human animal communication systems have indexical referential associations, i.e., they are based on a direct physical or temporal relation between sign-object or sign-event (Sinha, 2004; Nieder, 2009). The evolutionary transition from indexical communication in animals to symbolic communication in humans is considered to be associated with the emergence of language and symbolic thought (Deacon, 1998; Sinha, 2004; Nieder, 2009; Grouchy et al., 2016).

Even though only humans are thought to possess naturally occurring symbolic communication systems (i.e., natural languages, numerical systems), several other species such as apes, gray parrots, and dolphins can be trained to use symbols to express their needs/preferences when communicating with conspecifics (Fouts et al., 1984; Cianelli and Fouts, 1998; Pepperberg, 2009) or with humans (Gardner and Gardner, 1969; Herman et al., 1984; Schusterman and Krieger, 1984; Gisiner and Schusterman, 1992; Reiss and McCowan, 1993; Sevcik and Savage-Rumbaugh, 1994; Pepperberg, 2009). Symbolic communication between humans and animals can involve acoustic signals and speech (Herman et al., 1984; Pepperberg, 2009), gestures (Herman et al., 1984; Schusterman and Krieger, 1984, 1986), and technical interfaces such as TV monitors (Herman et al., 1990), interactive keyboards (Savage-Rumbaugh and Rumbaugh, 1978; Savage-Rumbaugh et al., 1980; Reiss and McCowan, 1993), or touchscreens (Nilsson et al., 2004; Amundin et al., 2008).

Training animals to communicate *via* arbitrary symbols has revealed astonishing mental capacities (Pepperberg, 1987, 2006; Boysen and Berntson, 1989; Reiss and McCowan, 1993; Savage-Rumbaugh and Fields, 2000; Kilian et al., 2003) which could have been overlooked if only the animals’ naturally occurring communication signals had been decoded. When animals communicate with humans *via* learned arbitrary symbols, sign-object and sign-event relations are much more common than sign-sign relations (Sevcik and Savage-Rumbaugh, 1994; Pepperberg, 2009). Nevertheless, this simple “symbolic” communication is highly useful for understanding which cognitive prerequisites were necessary for the evolution of true symbolic communication, i.e., language in humans. Moreover, it allows for an in-depth investigation of species-specific mental capacities. Researchers documented, for example, cognitive skills such as numerical competence (Boysen and Berntson, 1989; Pepperberg, 2006), concept formation (Pepperberg, 1987), associative learning capabilities, and self-organized learning events (Reiss and McCowan, 1993).

Here, we want to give our perspective on the potential capability of bats to communicate with humans by using arbitrary symbols. Albeit bats have not yet been trained to communicate *via* symbols with humans, we are convinced for reasons that we outline below, that they show the potential to master this cognitive task. Bats are a very gregarious taxon comprising >1,400 extant species and exhibit a large spectrum of social systems with differing degrees of complexity (Wilkinson et al., 2019). Because taxonomic breadth is crucial for studying cognitive adaptations and achievements (Dukas, 2004), bats are an important taxon for comparative cognition research. Many bat species are long-lived (up to 30 years in the wild; Barclay and Harder, 2003) and most species either live in perennial stable groups (Wilkinson and Boughman, 1998) or have a social organization characterized by fission-fusion dynamics (Kerth, 2008). Both forms of temporal consistency in social interactions between group members pose different requirements on the cognitive abilities of the animals because they differ considerably in terms of relevant group size, frequency of repeated encounters, and consistency of social relationships.

**Figure 1:**
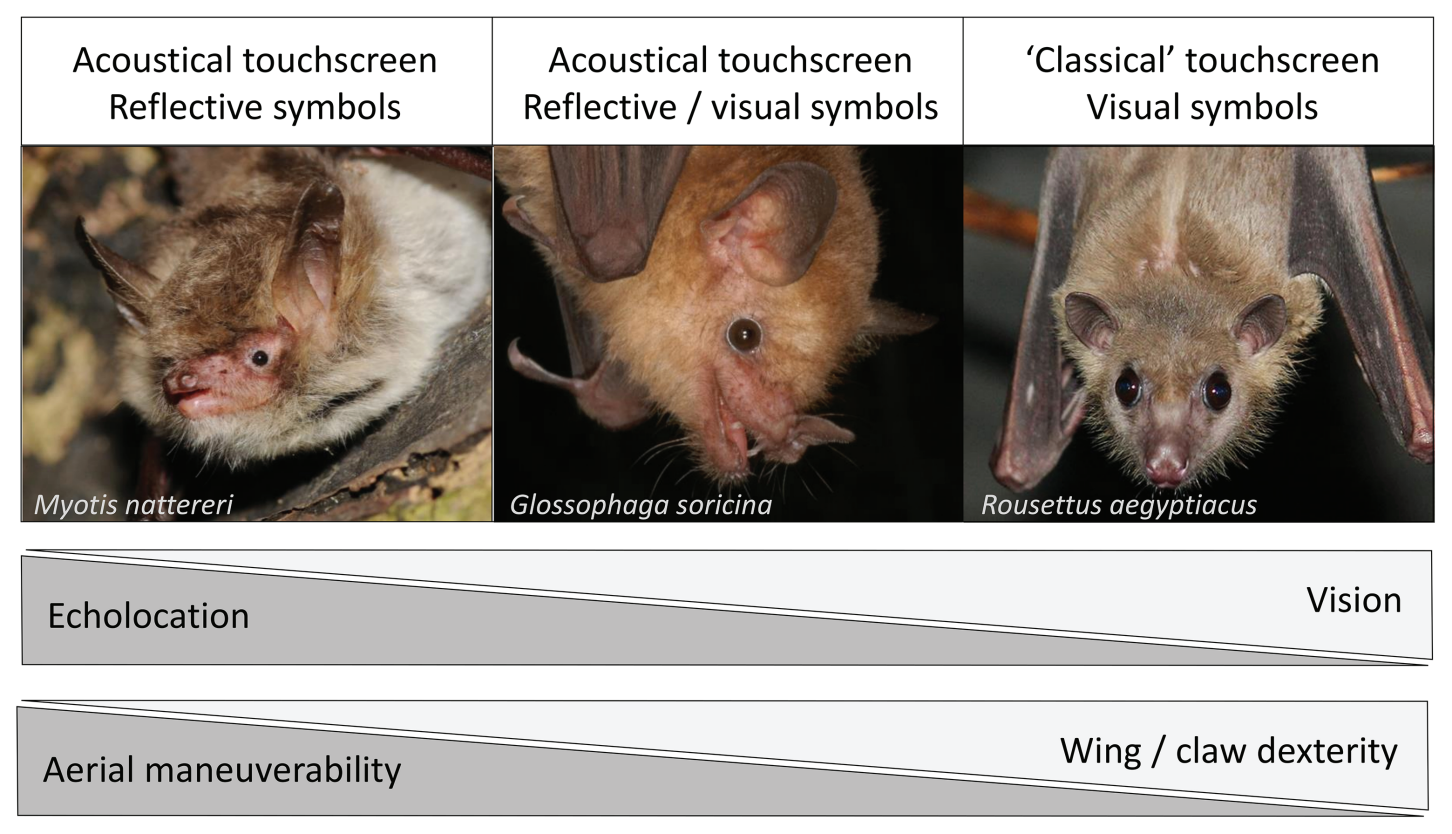
Knowledge about species-specific strength and weaknesses in perception, maneuverability, and dexterity must inform the training paradigms for bat-human communication, e.g., with a touchscreen. Whereas most bats rely on echolocation to perceive the world, many species also use vision to a certain degree. For the Pteropodid bats (“flying foxes”), vision is the most important sense and only some members of the genus *Rousettus* can use rudimentary echolocation based on tongue clicks. Whereas Pteropodid bats reach comparatively high levels of dexterity with their wings and claws and often use them to manipulate objects, many non-Pteropodid bats do not. In turn, non-Pteropodid bats generally show greater aerial maneuverability than Pteropodid bats. Thus, visually oriented bats with high dexterity should be trained to use a “classical” touchscreen with visual symbols which they can approach by crawling/climbing whereas echoacoustically oriented bats with high aerial maneuverability should be trained to use a touchscreen with reflective symbols which they can activate with their sonar beam while hovering in front of it. If necessary, intermediate forms of these two extremes should be used to best accommodate a species’ capabilities. The three depicted bat species represent the range of diverse species covered in the text: *Myotis nattereri*, an insectivorous gleaner (photo credit: Ján Svetlík), *Glossophaga soricina*, a nectarivorous flower-visiting bat (photo credit: Marco Tschapka), and *Rousettus aegyptiacus*, a frugivorous pteropodid (photo credit: Lithuanian Zoological Gardens).

Acoustic communication is one of the main channels for information transfer used by bats (Chaverri et al., 2018). In addition to echolocation (i.e., for navigation and foraging), different bat species possess diverse vocal repertoires and specific vocalization types which encode various information types such as emotional state (Bastian and Schmidt, 2008; Walter and Schnitzler, 2019) and identity information such as social group affiliation (Wilkinson and Boughman, 1998; Knörnschild et al., 2012), age (Jones et al., 1991; Fernandez and Knörnschild, 2017), and individual signatures (Carter et al., 2008; Chaverri et al., 2010). Vision and olfaction, the other two main sensory modalities in bats, are less well understood. Both phylogeny and species-specific dietary preferences influence bats’ visual capabilities (Figure 1): whereas most Old Word fruit bats (*Pteropodidae*) rely almost exclusively on vision for orientation (Möhres and Kulzer, 1956), only some members of the genus *Rousettus* can use rudimentary echolocation based on tongue clicks (Grinnell and Hagiwara, 1972). Acoustics are of crucial importance to insectivorous bats which capture their prey *via* echolocation (Neuweiler, 1989). In contrast to insectivorous bats, nectarivorous and frugivorous bats have comparably larger eyes and a better vision (Zhao et al., 2009), even though they predominantly rely on echolocation as well, especially at short range-distances (Winter et al., 2005; Holland, 2007). Olfaction plays an important additional role for foraging Pteropodids and frugivorous or nectarivorous Neotropical bats (Korine and Kalko, 2005; Raghuram et al., 2009; Gonzalez-Terrazas et al., 2016). Olfactory signals are also important mediators for social communication (Safi and Kerth, 2003; Voigt et al., 2008). However, bat olfaction will not be discussed further as this sensory modality is not well suited for training paradigms discussed later.

In the following, we briefly review what cognitive skills that constitute relevant prerequisites for symbolic communication are already known to be present in bats. Furthermore, we highlight potential training paradigms which could be used to elicit simple “symbolic” bat-human communication, i.e., bats using learned arbitrary symbols to elicit a desired behavior of the human caregiver. We hope to highlight practical approaches for future studies on symbolic communication in bats.

## Social Learning

Social learning occurs when animals learn from others that they observe or with whom they interact, for example, about foraging strategies or predator avoidance (Hoppitt and Laland, 2013). In bats, social learning is widespread and includes learning about roost‐ or food-related information as well as vocal production learning (reviewed in Wilkinson and Boughman, 1999; Wright, 2016). Learning from conspecifics has received much more attention than learning from heterospecific bats (Page and Bernal, 2020); the latter has been investigated in only a few species so far (Clarin et al., 2014; Patriquin et al., 2018). Moreover, the majority of studies demonstrated horizontal social learning, i.e., adults learning from adults, whereas vertical social learning, i.e., pups learning from adults, is currently understudied and yields both positive (Ripperger et al., 2019) and negative results (Rose et al., 2019). Although bats learn faster from other bats than from humans (Gaudet and Fenton, 1984; Clarin et al., 2014), humans can nevertheless elicit associative learning in bats and train them to perform specific actions (reviewed in Siemers and Page, 2009).

## Associative Learning

Bats readily learn to associate a particular cue with a specific outcome, either by themselves *via* trial-and-error learning or from others *via* social learning. Associative learning has been mainly demonstrated in a foraging context (reviewed in Wilkinson and Boughman, 1999; Wright, 2016). Bats can be trained to associate various novel cues with a food reward, e.g., light cues (Clarin et al., 2014), acoustic cues (Jones et al., 2013), echoacoustic, i.e., reflective cues (Simon et al., 2014), olfactory cues (Page et al., 2012), and visual cues (Manske and Schmidt, 1979). Gleaning bats, i.e., species that capture prey from substrates, seem to be especially well suited for food-related associative learning tasks (Siemers, 2001; Page and Ryan, 2006; Hulgard and Ratcliffe, 2014; Patriquin et al., 2018). Nectarivorous bats also exhibit strong associative learning in a foraging context and can be trained to discriminate fine-scale differences between sensory cues (von Helversen, 2004; Simon et al., 2006; Ross and Holderied, 2013) but they generally rely more on spatial cues than sensory cues (Thiele and Winter, 2005; Stich and Winter, 2006; Carter et al., 2010). Insectivorous bats can be trained to recognize 3-D objects as acoustic landmarks and associate them with safe passage through a net opening (Yu et al., 2019). In many species, learned associations are flexible and bats can be trained to reverse their initial associations (Page and Ryan, 2005; Clarin et al., 2013; Ross and Holderied, 2013). There is very little data on how long learned associations are remembered but current evidence suggests that bats have good short‐ and long-term memory (Ruczyński and Siemers, 2011; Page et al., 2012; Clarin et al., 2014; but see: Hernández-Montero et al., 2020). The above-mentioned examples used positive reinforcement but associative learning can also be negatively reinforced. Bats readily acquire taste aversions, e.g., by associating a novel acoustic cue with a noxious food reward (Bates and Fenton, 1990) or a novel flavor cue with an episode of toxicosis (Ratcliffe et al., 2003).

## Trainability by Humans

Various techniques can be applied to coax bats to participate in associative learning tasks (reviewed in Siemers and Page, 2009). Two important techniques for training bats are fading and shaping (Terrace, 1963; Shettleworth, 1998; Domjan, 2003). When fading, bats are gradually introduced to a new stimulus by altering the stimulus in small steps (Jones et al., 2013; Hemingway et al., 2020). Fading is especially important when studying reversal learning as it also allows the removal of a bat’s response to a known stimulus (Page and Ryan, 2005, 2006). When shaping, the desired response of a bat is increasingly reinforced while non-desired responses are not reinforced (Barber et al., 2003). Shaping is also the technique of choice when training bats to perform certain behaviors on command. Captive Pteropodid bats (“flying foxes”) can be readily trained for husbandry and vet checks; for instance, they can learn to follow a target, to unfold their wings in response to a hand signal, and to touch an item on demand (pers. communication Brian Pope, Lubee Bat Conservancy, USA). We are not aware that non-Pteropodid bats are being trained for husbandry and vet checks. However, temporarily captive non-Pteropodid bats can be trained to approach humans to retrieve a food reward, to wait on a perch until the onset of a stimulus, and to fly to a specific position when perceiving a stimulus (Tuttle, 2019).

## Imitation

Several bat species are capable of imitating conspecifics’ actions. Naïve individuals have been shown to learn about novel foraging situations by paying close attention to knowledgeable conspecifics (*Eptesicus fuscus*: Wright et al., 2011; *Antrozous pallidus*: Bunkley and Barber, 2014). Imitation has also been shown in a communicative context, namely, when pups learn to sing by imitating the song of adult tutors (*Saccopteryx bilineata*: Knörnschild et al., 2010).

## Vocal Production Learning and Usage Learning

Imitating new signals is one form of vocal production learning (VPL), modifying existing signals based on social influences is another (Janik and Slater, 1997, 2000). VPL *via* social modification has been shown for social calls (*Rousettus aegyptiacus*: Prat et al., 2015, 2017; Genzel et al., 2019; *Saccopteryx bilineata*: Knörnschild et al., 2012; *Phyllostomus discolor*: Esser and Schmidt, 1989; Esser, 1998; Lattenkamp et al., 2020; *P. hastatus*: Boughman, 1998) and echolocation calls (*Rhinolophus ferrumequinum*: Jones and Ransome, 1993; *Hipposideros terasensis*: Hiryu et al., 2006). In addition to VPL, vocal usage learning has been demonstrated by training temporarily isolated bats to vocalize in order to trigger a food reward (*P. discolor*: Lattenkamp et al., 2018). It is plausible that more bat species may have some degree of volitional control over their vocalizations but data are currently lacking.

## Social Knowledge

Social knowledge describes the cognitive assessment of cues that communicate socially relevant information (Cheney et al., 1986). Whereas social knowledge mainly constitutes learning about others, such as their status or intentions, sociocognitive skills also facilitate the interpretation of signals or cues from others outside a social context (e.g., using gaze following to identify the location of food that a conspecific has hidden; Tomasello et al., 1998). In bats, social knowledge is severely understudied and most circumstantial evidence concerns comparatively simple sociocognitive skills such as the maintenance of dominance hierarchies (Neuweiler, 1969) or territorial interactions (Voigt and Streich, 2003). Advanced sociocognitive skills such as gaze following, joint attention, point following, and theory of mind are found to varying degrees in highly intelligent social species, such as primates and corvids, and also in domesticated species such as dogs; they can include heterospecific interactions, for example with humans (reviewed in Fitch et al., 2010). Evidence for heterospecific social knowledge in bats is currently limited to one study which demonstrated that captive born individuals of different bat species (*Pteropus pumilus*, *P. rodricensis*, and *P. conspicillatus*) are responsive to human pointing gestures (Hall et al., 2011): experimentally naïve bats readily utilize human pointing to find the location of concealed food in an object-choice task. The observed spontaneous point-following behavior suggests advanced sociocognitive skills in these bats. Interestingly, only captive born individuals were sensitive to human gestures; captive individuals born in the wild (*P. pumilus* and *P. vampyrus*) were not (Hall et al., 2011). It is possible that direct contact with humans early in ontogeny is necessary for bats to exhibit heterospecific point-following behavior.

## Discussion

There is conclusive evidence, albeit sometimes anecdotal, that different bat species possess several key prerequisites necessary for symbolic communication, most importantly associative learning and a general readiness to interact with and learn from caregivers in captivity. However, it is important to note that the ability for associative learning alone is not a guarantee that bats can transfer simple associations to more complex symbolic representations. What is missing so far is an experimental approach that actively combines these abilities to test if rudimentary symbolic bat-human communication can be achieved.

**Figure 2:**
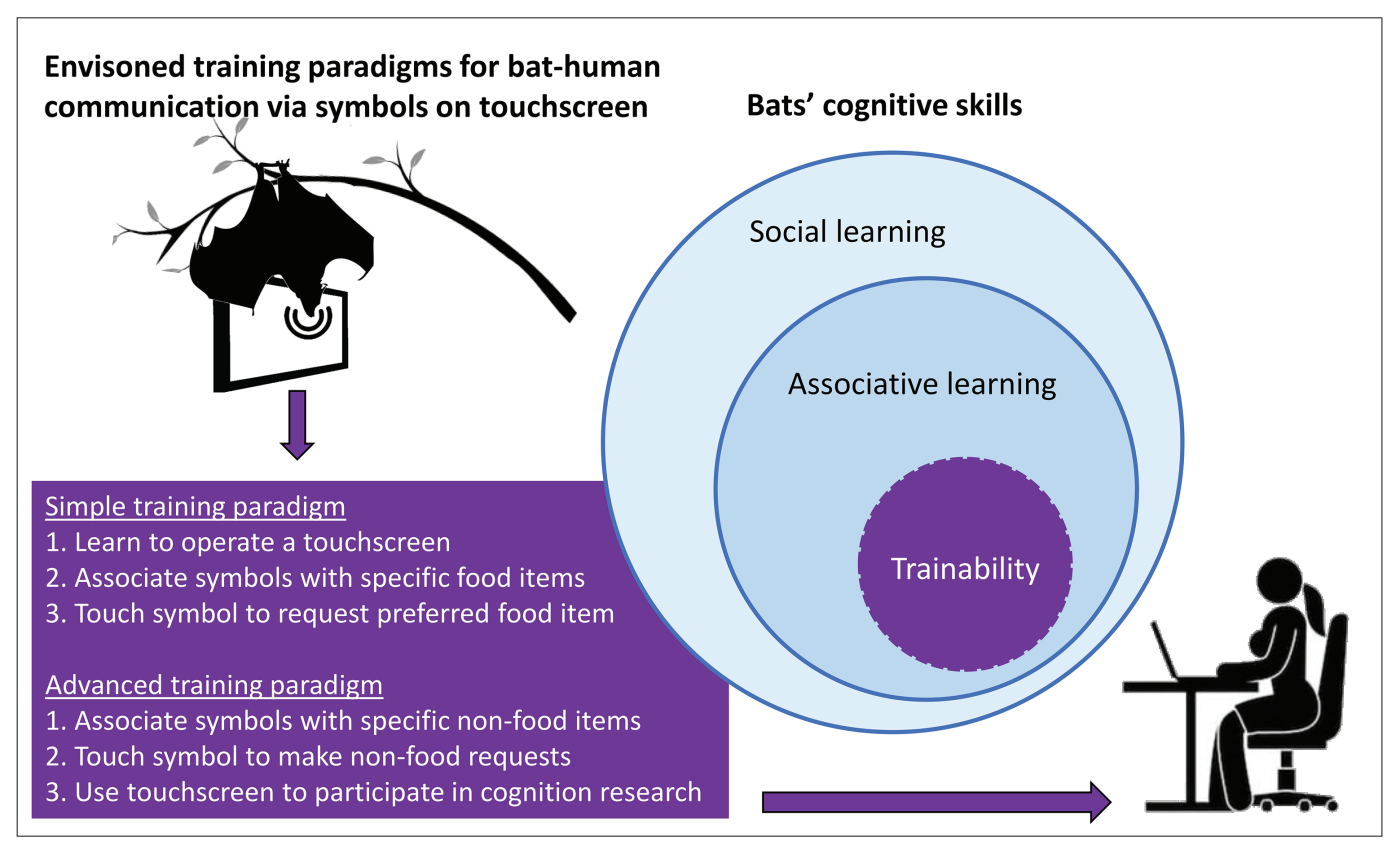
Envisioned training paradigms for bat-human communication *via* symbols on a touchscreen. A set of different cognitive skills should enable bats to use a touchscreen, most importantly their capability of associative learning in a social context. We suggest focusing on visual or echoacoustic, i.e., reflective symbols on a touchscreen. A simple training paradigm requires bats to learn to operate a touchscreen by touching visual symbols with their snout (or reflective symbols with their sonar beam), to associate different symbols with specific food items, and to use these symbols to communicate which food item they prefer to receive (sign-object relation). An advanced training paradigm requires bats to associate different symbols with specific non-food items, e.g., caresses, access to toys, etc., and to use these symbols to communicate their preference (sign-event or sign-object relation). Touchscreen-proficient bats can participate in cognition research, e.g., to study their numerical competence or categorical perception.

If attempted, we suggest making the task as easy as possible in both implementation and perception to facilitate the initial communication process. Training bats to communicate their choice between different preferred food items *via* arbitrary symbols would be a promising starting point to implement bat-human communication. Touchscreens are very promising tools for animal-human communication because they can be activated *via* fingers, snouts, tongues, beaks, and sonar beams, thus, making them accessible to a wide range of taxa (reviewed in Egelkamp and Ross, 2019). Bats would need (1) to learn to operate a touchscreen, (2) learn the association of a certain symbol with a specific food item, and (3) to use the symbol when communicating with a human *via* a touchscreen (Figure 2).

Accommodating species-specific differences in perception is crucial for the success of this endeavor (Figure 1). Visually oriented bats such as Pteropodids could be trained to use a touchscreen with visual symbols representing different preferred food items, as has been successfully done with primates (Savage-Rumbaugh, 1993). Echoacoustically oriented bats could be trained to use an acoustically activated touchscreen instead. This method, termed Echo Location Visualization and Interface System (ELVIS), has been developed for dolphins (Nilsson et al., 2004; Amundin et al., 2008) and allows them to use their sonar beam to “touch” and, thus, choose items on a screen, e.g., to communicate food preferences (Starkhammar et al., 2007). For bats, an acoustically activated touchscreen would ideally not depict visual symbols but reflective symbols (e.g., reliefs) to facilitate perception.

Even though bats are capable of vocal production and usage learning, we would advise against the use of acoustic symbols to facilitate bat-human communication. In contrast to certain songbirds, parrots, and dolphins, the imitation of heterospecific sounds has never been demonstrated in bats. Because heterospecific vocal imitation is crucial for using novel sounds as symbols, we suggest focusing on visual or echoacoustic, i.e., reflective symbols for bat-human communication instead.

To conclude, bats are a promising taxon for future studies on symbolic communication with humans. Their willingness to interact with caregivers, associative learning abilities, and advanced (socio-)cognitive skills are important prerequisites to communicate successfully with humans. If bat-human communication about food requests could indeed be established, it would be an ideal stepping stone for a more advanced comparative cognition research, further elucidating how nonhuman animals think and learn.

## Author Contributions

MK and AF reviewed the literature and wrote the manuscript. Both the authors contributed to the article and approved the submitted version.

### Conflict of Interest

The authors declare that the research was conducted in the absence of any commercial or financial relationships that could be construed as a potential conflict of interest.
